# Proteomic profiling of bone tissue reveals distinct pathways in men and women with osteoporosis

**DOI:** 10.1093/ageing/afaf299

**Published:** 2025-10-17

**Authors:** Xiaoyun Lin, Jing Hu, Hengyan Zhang, Lei Sun, Bingna Zhou, Yan Jiang, Ou Wang, Weibo Xia, Jia Zhang, Mei Li

**Affiliations:** Department of Endocrinology, National Health Commission Key Laboratory of Endocrinology, Peking Union Medical College Hospital, Chinese Academy of Medical Sciences & Peking Union Medical College, Beijing, China; Department of Endocrinology, Fujian Provincial Hospital, Shengli Clinical Medical College of Fujian Medical University, Fuzhou University Affiliated Provincial Hospital, Fuzhou, China; Department of Endocrinology, National Health Commission Key Laboratory of Endocrinology, Peking Union Medical College Hospital, Chinese Academy of Medical Sciences & Peking Union Medical College, Beijing, China; Department of Orthopedics, Peking Union Medical College Hospital, Chinese Academy of Medical Sciences & Peking Union Medical College, Beijing, China; Department of Endocrinology, National Health Commission Key Laboratory of Endocrinology, Peking Union Medical College Hospital, Chinese Academy of Medical Sciences & Peking Union Medical College, Beijing, China; Department of Endocrinology, National Health Commission Key Laboratory of Endocrinology, Peking Union Medical College Hospital, Chinese Academy of Medical Sciences & Peking Union Medical College, Beijing, China; Department of Endocrinology, National Health Commission Key Laboratory of Endocrinology, Peking Union Medical College Hospital, Chinese Academy of Medical Sciences & Peking Union Medical College, Beijing, China; Department of Endocrinology, National Health Commission Key Laboratory of Endocrinology, Peking Union Medical College Hospital, Chinese Academy of Medical Sciences & Peking Union Medical College, Beijing, China; Department of Endocrinology, National Health Commission Key Laboratory of Endocrinology, Peking Union Medical College Hospital, Chinese Academy of Medical Sciences & Peking Union Medical College, Beijing, China; Department of Orthopedics, Peking Union Medical College Hospital, Chinese Academy of Medical Sciences & Peking Union Medical College, Beijing, China; Department of Endocrinology, National Health Commission Key Laboratory of Endocrinology, Peking Union Medical College Hospital, Chinese Academy of Medical Sciences & Peking Union Medical College, Beijing, China

**Keywords:** osteoporosis, proteomics, immunoinflammatory response, oxidative stress, older people

## Abstract

**Background:**

Osteoporosis is characterised by an imbalance in bone remodelling, yet the detailed molecular mechanisms underlying its pathogenesis remain unclear. This study aimed to identify key proteins and regulatory pathways associated with severe osteoporosis in women and men.

**Methods:**

Bone specimens were collected during surgery from 13 women and 12 men with osteoporotic fractures, along with 8 female and 7 male controls with violent fractures. Differentially abundant proteins (DAPs) in bone tissues were identified via nontargeted liquid chromatography–tandem mass spectrometry proteomics. Functional enrichment and pathway analyses were performed; the diagnostic potential of core DAPs was evaluated through multivariate receiver operating characteristic (ROC) analysis, and correlations between DAPs and clinical parameters were assessed.

**Results:**

DAPs of women with osteoporotic fractures were primarily associated with immunoinflammatory response, while DAPs of men with osteoporotic fractures were predominantly related to oxidative stress. ROC analysis revealed strong associations between core upregulated proteins and osteoporosis in both women [area under the ROC curve (AUC) = 0.908, 95% confidence interval (CI): 0.676–1] and men (AUC = 0.851, 95% CI: 0.366–1). After adjusting for age, HLA-C in women was significantly negatively correlated with bone mineral density (*P* < .05), while NDUFA11, COX7A2, NDUFAB1, UQCRC1, COX5A and VDAC1 in men were significantly positively correlated with β-C-terminal telopeptide of type I collagen (*P* < .05).

**Conclusion:**

This study identified sex-specific proteomic profiles and molecular pathways associated with osteoporosis, with ageing emerging as a central upstream driver of these differences. These findings may help elucidate the sex differences in the pathogenesis of osteoporosis and provides a foundation for the discovery of new therapeutic targets and the development of personalised precision treatment strategies.

## Key Points

- Osteoporosis in women and men exhibited different proteomic features, involving distinct biological functions and pathways.
- Differentially abundant proteins (DAPs) in women with osteoporotic fractures were related to immunoinflammatory response pathways.
- DAPs in men with osteoporotic fractures were associated with oxidative stress response pathways.
- Significant correlations were found between core upregulated proteins and osteoporosis in both women and men.

## Introduction

Osteoporosis is an ageing-related skeletal disorder characterised by disrupted bone remodelling processes, where bone resorption exceeds bone formation, leading to decreased bone mass, deterioration of bone microarchitecture and increased susceptibility to fractures [1]. Despite advancements in understanding bone metabolism, the in-depth molecular mechanisms by which bone remodelling imbalance leads to osteoporosis are still not well understood.

Proteomics offers a powerful approach to identify key proteins and signalling pathways involved in bone metabolism [2]. Previous studies using serum or monocyte proteomics methods have identified several factors involved in osteoporosis; however, the results have been inconsistent [3, 4]. Moreover, as the samples in most studies were derived from blood, the differentially abundant proteins (DAPs) may not adequately reflect the localised inflammatory processes after osteoporotic fractures [5], nor can they be definitively related to the pathogenesis of osteoporotic fractures. Proteomic studies on human bone samples are extremely rare, which can more directly capture the skeletal intrinsic pathological status underlying osteoporosis.

Additionally, although osteoporosis significantly affects both women and men—with osteoporotic fractures occurring in one in three women and one in five men over 50 years old among the global population [6]—the majority of proteomic research has focused on osteoporosis in women, while research on male osteoporosis remains limited [7], particularly bone tissue proteomic studies specific to males. Furthermore, previous research has identified sex-related differences in osteoporosis, such as variations in bone loss rates and preferred anatomical sites of bone deterioration [8]. However, it remains unclear whether there are distinct molecular mechanisms underlying osteoporosis between women and men.

Therefore, this study aimed to investigate the core proteins and signalling regulatory pathways associated with severe osteoporosis by comparing bone tissue proteomic profiles between patients with osteoporotic fractures and sex-matched controls with violent fractures, in both women and men.

## Methods

### Subjects

Patients were recruited prospectively between September 2021 and June 2023 from those admitted to the Orthopaedic Department of Peking Union Medical College Hospital (PUMCH) . The flow chart of patient recruitment was illustrated in Supplementary Figure S1.Patients with fragility fractures (severe osteoporosis group) and sex-matched subjects with violent fractures (control group) were included. Inclusion criteria for severe osteoporosis were postmenopausal women or men ≥50 years with fragility fractures (hip, vertebrae, proximal humerus, pelvis or distal forearm) and bone mineral density (BMD) T-score < −1.0. Controls were sex-matched patients with violent fractures (hip, vertebrae, proximal humerus, pelvis, distal forearm, tibia/fibula, patella, or calcaneus) and BMD T-score ≥ −1.0 for participants ≥50 and *Z*-score > −2.0 for those <50. Patients with conditions affecting bone metabolism (malignant tumours, hyperparathyroidism, hyperthyroidism, Cushing’s syndrome, Parkinson’s disease, diabetes mellitus, chronic obstructive pulmonary disease, chronic liver or renal failure) or receiving medications affecting bone metabolism (anti-osteoporotic treatments within 12 months, glucocorticoids, chemotherapy, thyroid hormones, antiviral drugs, etc.) were excluded.

This study was approved by the Ethics Committee of PUMCH (JS-2798) and was conducted in accordance with the Declaration of Helsinki. Informed consent was obtained from all patients before participation.

### Clinical information collection

Clinical data were recorded, including age, sex, height, weight, cause and site of fracture, comorbidities and medication history. Fasting blood samples were collected between 8:00 and 10:00 a.m. before surgery for serum analyses including β-C-terminal telopeptide of type I collagen (β-CTX), procollagen type I N-terminal propeptide (P1NP), 25-hydroxyvitamin D (25OHD), parathyroid hormone (PTH), alanine aminotransferase (ALT), creatinine (Cr), calcium (Ca), phosphate (P) and alkaline phosphatase (ALP). BMDs at lumbar spine (LS), femoral neck (FN) and total hip (TH) were measured by dual-energy X-ray absorptiometry (DXA, Prodigy Advance, GE Healthcare) and standardised to T/Z scores according to the manufacturer’s standard procedures. Skeletal X-rays were performed to evaluate the fractures.

### Sample preparation for liquid chromatography–tandem mass spectrometry

Bone tissue specimens from patients with osteoporotic or violent fractures were collected intraoperatively. All surgeries were performed by the same surgical team to ensure consistency. For vertebral fractures, cancellous bone was obtained directly from the compressed vertebra during vertebral augmentation therapy. For non-vertebral fractures, bone tissue was harvested from the fracture site. All specimens were immediately rinsed in phosphate-buffered saline to remove debris, soft tissue and fat. The samples were homogenised in radio immunoprecipitation assay buffer containing protease inhibitors, lysed and centrifuged at 25 000 × g for 15 min. Protein supernatants were sequentially treated with 10 mM dithiothreitol (37°C, 30 min) and 55 mM iodoacetamide (room temperature, 45 min, dark). Protein concentrations were measured by Bradford assay. For quality control, 10 μg of protein was subjected to SDS–PAGE and Coomassie blue staining. Subsequently, 100 μg of protein per sample was digested with 2.5 μg trypsin (37°C, 4 h), desalted (Strata X column) and dried under vacuum.

### High-pH reversed-phase separation

Peptides were separated using a Shimadzu LC-20AB HPLC with a Gemini high-pH C18 column (5 μm, 4.6 × 250 mm). The sample was eluted at 1 ml/min with the gradient: 5% mobile phase B (95% acetonitrile, pH 9.8) for 10 min, 5% to 35% mobile phase B for 40 min, 35% to 95% mobile phase B for 1 min, held at 95% for 3 min and equilibrated at 5% for 10 min. Eluate was monitored at 214 nm; fractions were collected every minute, combined into 10 pools and freeze-dried.

### LC–MS/MS analysis

Dried fractions were reconstituted and analysed on an Orbitrap Fusion Lumos (Thermo Fisher Scientific) equipped with an EASY-nLC 1200. Peptides were loaded onto a C18 analytical column (1.8 μm, 150 μm × 35 cm) and eluted with 2%–32% mobile phase B (98% acetonitrile/0.1% formic acid) over 180 min at 500 nl/min.

For data-dependent acquisition (DDA): spray voltage 2 kV; MS^1^ range 350–1500 *m*/*z*, resolution 60 000, maximum injection time (MIT) 50 ms; each full scan was followed by 20 MS^2^ scans (resolution 15 000, automatic gain control [AGC] 2 × 10^4^, MIT 50 ms, dynamic exclusion 30 s).

For data-independent acquisition (DIA): MS^1^ range 400–1500 *m*/*z*, resolution 60 000, MIT 50 ms; one full scan was followed by 44 variable windows of 16 *m*/*z* width, higher-energy collisional dissociation 30%, MS^2^ resolution 30 000 (at *m*/*z* 200), AGC 5 × 10^4^, MIT 50 ms.

### LC–MS/MS data processing and analysis

Raw spectra were processed in MaxQuant (v 2.6.4.0) with Andromeda. For DIA runs, retention-time calibration was performed with iRT peptides, and a false-discovery rate (FDR) ≤ 1% was applied at both peptide and protein levels. Mass-error tolerances were set to 4.5 ppm for MS^1^ and 20 ppm for MS^2^ spectra. Protein intensities were normalised with the MSstats R package (v 4.2.0). DAPs were defined as those with a fold-change (FC) > 2.0 or < 0.5 and an FDR-adjusted *P* < .05 (Benjamini–Hochberg correction). Functional enrichment analysis was carried out in the DAVID database. Protein–protein interaction (PPI) networks were constructed in STRING. To assess the diagnostic value of core DAPs, multivariate receiver operating characteristic (ROC) analysis was performed with a support vector machine (SVM) algorithm in MetaboAnalyst 6.0 and cross-validated with partial least squares (PLS) and random forest models.

### Statistical analysis

The Kolmogorov–Smirnov test was used to assess the normal distribution of continuous variables. Normally distributed data, including age, body mass index (BMI), BMD, serum P1NP, β-CTX, ALT and Cr were expressed as the means ± standard deviations (SDs). Independent *t* tests were used to compare the differences in age, BMI, BMD and bone turnover biomarkers (BTMs) between groups. Qualitative data, including comorbidities and previous treatment history, were expressed as frequencies (%) and were compared between the two groups by Fisher’s exact test. The associations between core DAPs and BMD/BTMs were evaluated using partial correlation analysis to adjust for age as a confounding variable.

Statistical analyses were performed using SPSS software (version 26.0; SPSS Inc., Chicago, USA). A two-tailed value of *P* < .05 was considered statistically significant.

## Results

### Clinical characteristics of patients with osteoporotic or violent fractures

A total of 13 women with osteoporotic fractures, 8 women with violent fractures, 12 men with osteoporotic fractures and 7 men with violent fractures were included in this study. The demographic and clinical characteristics of these patients are shown in Table 1. Patients with osteoporotic fractures were significantly older than those with violent fractures (*P* < .001) and had lower BMD in the LS, FN and TH (*P* < .01). Among women with osteoporotic fractures, the serum levels of P1NP, ALP, 25OHD, Cr and ALT were significantly greater than in those with violent fractures (*P* < .05). Among men with osteoporotic fractures, height and BMI were significantly lower, and the serum P1NP level was significantly greater than in those with violent fractures (all *P* < .05). Fracture sites are detailed in Supplementary Table S1.

**Table 1:** Demographic and clinical characteristics of women and men with osteoporotic or violent fractures.

	Women			Men		
	Osteoporotic fracture (*n* = 13)	Violent fractures (*n* = 8)	*P* value	Osteoporotic fracture (*n* = 12)	Violent fractures (*n* = 7)	*P* value
Age, years	75.69 ± 12.22	33.75 ± 7.36	**<.001**	77.83 ± 8.29	52.00 ± 19.20	**.011**
Height, cm	155.88 ± 7.41	163.17 ± 6.08	.052	169.75 ± 7.25	177.00 ± 5.54	**.036**
Weight, kg	53.02 ± 8.59	58.42 ± 6.67	.193	65.04 ± 13.31	81.29 ± 4.23	**.002**
BMI, kg/m^2^	21.73 ± 2.31	21.95 ± 2.39	.850	22.63 ± 4.60	25.96 ± 1.26	**.034**
Comorbidities, *n* (%)						
Hypertension	4 (30.8)	1 (12.5)	.606	5 (41.7)	2 (28.6)	.656
Hyperlipidemia	7 (53.8)	0	**.018**	2 (16.7)	1 (14.3)	1.000
Coronary heart disease	5 (38.5)	0	.111	–	–	–
Benign prostatic hyperplasia	–	–	–	3 (25.0)	1 (14.3)	1.000
Hypothyroidism	–	–	–	2 (16.7)	0	.509
Others	4 (30.8)	2 (25.0)	1.000	2 (16.7)	1 (14.3)	1.000
Previous treatment history, *n* (%)						
Lipid-lowering medication	4 (30.8)	0	.131	2 (16.7)	1 (14.3)	1.000
Hypotensive drugs	4 (30.8)	1 (12.5)	.606	5 (41.7)	2 (28.6)	.656
Medication for prostatic hyperplasia	–	–	–	3 (25.0)	1 (14.3)	1.000
Others	1 (7.7)	1 (12.5)	1.000	2 (16.7)	0	.509
BMD, g/cm^2^						
LS	0.79 ± 0.10	1.19 ± 0.14	**<.001**	0.89 ± 0.11	1.21 ± 0.18	**<.001**
FN	0.63 ± 0.09	0.90 ± 0.11	**<.001**	0.74 ± 0.10	0.95 ± 0.10	**<.001**
TH	0.68 ± 0.10	0.96 ± 0.14	**<.001**	0.80 ± 0.12	1.01 ± 0.12	**.003**
P1NP, ng/ml	75.08 ± 30.73	43.35 ± 15.84	**.035**	90.29 ± 44.97	43.45 ± 20.66	**.010**
β-CTX, ng/ml	0.75 ± 0.30	0.60 ± 0.19	.281	0.67 ± 0.24	0.56 ± 0.15	.326
PTH, pg/ml	36.97 ± 15.90	36.27 ± 10.47	.925	46.81 ± 32.57	36.85 ± 11.77	.486
25OHD, ng/ml	23.33 ± 9.28	10.10 ± 2.39	**.004**	16.70 ± 7.62	13.68 ± 6.53	.427
Ca, mmol/L	2.26 ± 0.09	2.21 ± 0.03	.116	2.20 ± 0.14	2.25 ± 0.05	.321
P, mmol/L	1.20 ± 0.21	1.40 ± 0.10	.081	1.02 ± 0.13	1.12 ± 0.13	.248
ALP, U/L	105.56 ± 30.15	62.80 ± 17.48	**.014**	103.36 ± 32.49	85.25 ± 36.94	.372
Cr, μmol/L	63.85 ± 12.44	48.75 ± 10.67	**.010**	72.58 ± 15.45	76.14 ± 12.94	.615
ALT, U/L	13.46 ± 4.16	9.38 ± 3.29	**.029**	16.25 ± 12.84	25.00 ± 10.89	.149

Normally distributed data are presented as mean ± SD.

Categorical data are expressed as frequencies (%).

25OHD, 25-hydroxyvitamin D; ALP, alkaline phosphatase; ALT, glutamic-pyruvic transaminase; BMD, bone mineral density; BMI, body mass index; Ca, calcium; Cr, creatinine; FN, femoral neck; LS, lumbar spine; P, phosphorus; P1NP, procollagen type 1 N-peptide; PTH, parathyroid hormone; β-CTX, β-C-terminal telopeptide of type 1 collagen.

Bold numbers represent *P* < .05.

### Identification of DAPs in patients with osteoporotic or violent fractures

**Figure 1:**
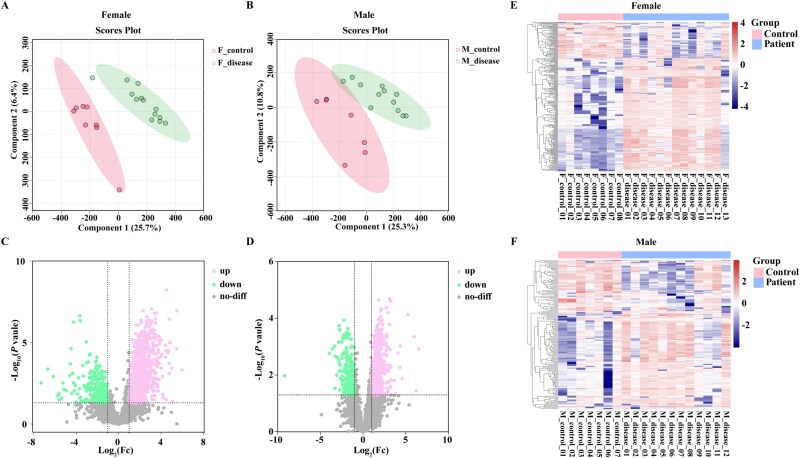
PLS-DA, volcano plot and heatmap of DAPs from women and men with osteoporotic or violent fractures. (A–B) PLS-DA plots of DAPs in women (A) and men (B) with osteoporotic fractures versus violent fractures . (C–D) Volcano plots of up-regulated and down-regulated proteins in women (C) and men (D). (E–F) Hierarchical clustering heatmaps of DAPs in women (E) and men (F) with osteoporotic fractures (blue) versus violent fractures (red) DAPs; PLS-DA, partial least squares discriminant analysis; up, upregulated; down, downregulated; no-diff, no difference.

Quantitative proteomic analysis of bone tissues identified 66 365 peptides corresponding to 6045 proteins. Initial principal component analysis did not separate groups clearly (see Supplementary Figure S2 in the Supplementary Data). Subsequent supervised dimensionality reduction using partial least squares discriminant analysis clearly distinguished the severe osteoporosis group from controls in both women and men (Figure 1A and B).

In the female osteoporosis-control comparison group, 1161 DAPs were identified, with 917 proteins upregulated and 244 downregulated in the osteoporosis group (Figure 1C). In the male osteoporosis-control comparison group, 786 DAPs were identified, with 560 proteins upregulated and 226 downregulated in the osteoporosis group (Figure 1D).

### Pathway enrichment analysis

To refine the data, proteins with missing expression values ≥50% in each group were removed. For the female comparison group, we further screened for DAPs with an Fc ≥ 5.0 or ≤ 0.2, identifying 185 upregulated and 60 downregulated proteins. Similarly, for the male comparison group, we screened for DAPs with an Fc ≥ 3.0 or ≤ 0.2, identifying 95 upregulated and 60 downregulated proteins. Hierarchical clustering analysis revealed significant differences in protein expression between patients with osteoporotic fractures and violent fractures in both women and men (Figure 1E and F).

**Figure 2:**
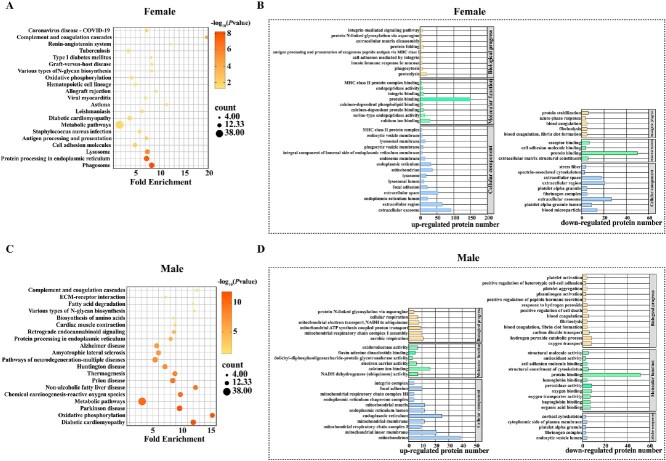
KEGG pathway and GO enrichment analyses of DAPs in women and men. (A and C) KEGG pathway enrichment analyses of selected DAPs in women (A) and men (C). (B and D) GO analyses of biological processes , molecular functions and cellular components for DAPs in women (B) and men (D). DAPs; GO, Gene Ontology; KEGG, Kyoto Encyclopedia of Genes and Genomes.

KEGG pathway enrichment analysis revealed that the DAPs in women were enriched mainly in phagosomes, lysosomes, cell adhesion molecules, endoplasmic reticulum protein processing, antigen processing and presentation, and metabolic pathways (Figure 2A). GO functional annotation analysis indicated that these proteins were involved primarily in biological processes such as protein hydrolysis, phagocytosis, the mucous membrane innate immune response, integrin-mediated cell adhesion, antigen processing and MHC class I presentation, which are associated with the immunoinflammatory response (Figure 2B).

Together, these processes are associated with immune dysfunction and inflammation, suggesting a potential link to the immunoinflammatory response in postmenopausal osteoporosis.

In men, KEGG enrichment analysis of the top 155 DAPs revealed that these proteins were related mainly to oxidative phosphorylation, metabolic pathways, reactive oxygen species (ROS) and endoplasmic reticulum protein processing pathways (Figure 2C). GO analysis indicated that these proteins were involved primarily in the mitochondrial respiratory chain and related biological processes (Figure 2D). Therefore, mitochondrial dysfunction in men could lead to energy deficits and the accumulation of ROS, contributing to oxidative stress and bone cell damage. These findings suggest a potential link between oxidative stress activation and the pathogenesis of male osteoporosis.

### Identification of core DAPs

We discovered 9 proteins in women and 10 in men that were significantly expressed in each KEGG pathway. PPI network analysis indicated significant interactions (see Supplementary Figure S3 in the Supplementary Data). Further investigation via the Monarch Initiative database revealed that 7 core DAPs (ATP6V0A1, HLA-DRB1, HLA-C, HLA-DRA, HLA-A, ITGB2 and HLA-DPB1) in women were strongly associated with ‘bone measurement (EFO:0004512)’ (association strength: 0.82; false positive rate: 0.0497), which refers to measurable bone traits such as BMD, bone size and bone geometry. Further analysis via the Human Protein Atlas database revealed that the 10 DAPs in men are expressed in bone marrow and skeletal muscle tissues, suggesting that they may contribute to bone loss and increased fracture risk by affecting bone and muscle function (see Supplementary Table S2 in the Supplementary Data).

**Figure 3:**
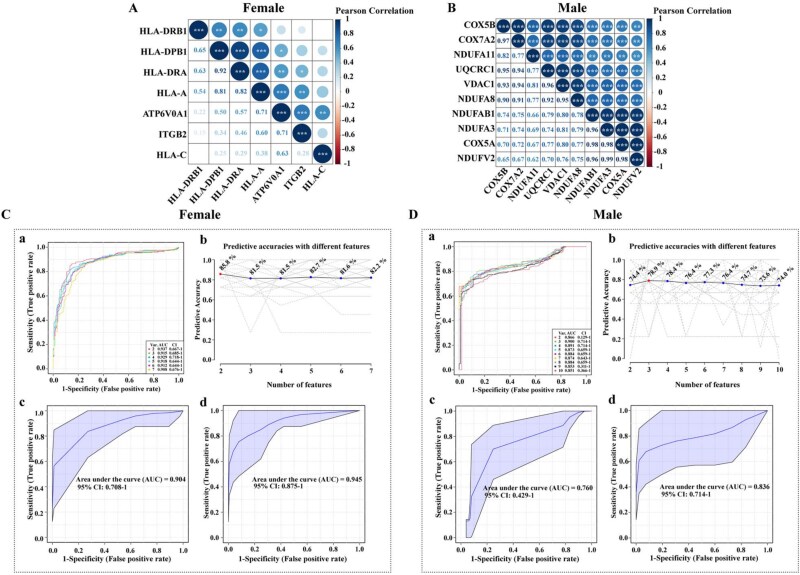
Correlation and ROC analyses of core DAPs in women and men. (A–B) Correlation heatmaps of core DAPs in women (A, 7 proteins) and men. (B, 10 proteins). ^***^P < .001, ^**^P < .01, ^*^P < .05. (C–D) ROC curves evaluating the performance of core DAPs to distinguish osteoporotic from violent fractures in women (C) and men (D) using SVM, PLS-DA and random forest models. AUC, area under the ROC curve; CI, confidence interval; DAPs; PLS-DA, partial least squares discriminant analysis; ROC, receiver operating characteristic; SVM, support vector machine.

Correlation analyses revealed significant intercorrelations among these core proteins within each sex (Figure 3A and B), indicating overlapping pathways in the pathogenesis of osteoporosis. Specifically, in women, HLA-DRA and HLA-DPB1 presented the strongest correlation (*r* = 0.92, *P* < .001) (Figure 3A). In men, NDUFV2 and NDUFA3 exhibited the strongest correlation (*r* = 0.99, *P* < .001) (Figure 3B). These findings suggested that, in women, HLA-DRA and HLA-DPB1 might be involved in the same pathway or biological process, and, in men, NDUFV2 and NDUFA3 might also be involved in the same pathway or biological process.

### Associations between core DAPs and osteoporosis

In women, SVM analysis yielded an area under the ROC curve (AUC) of 0.908 (accuracy 82.2%; Figure 3C.a, b) for the 7 core DAPs. Validation via PLS-DA and random forest models showed AUCs of 0.904 and 0.945, respectively, indicating robust associations between these 7 core DAPs and osteoporosis (Figure 3C.c, d). In men, SVM analysis yielded an AUC of 0.851 for the 10 core DAPs (accuracy 74%; Figure 3D.a, b), validated by PLS-DA (AUC = 0.76) and random forest (AUC = 0.836), suggesting a moderate to strong association between the 10 core DAPs and osteoporosis (Figure 3D.c, d).

Additionally, we explored the correlation between the relative expression levels of core DAPs and BMDs and BTMs. These core proteins showed significant negative correlations with BMD (*P* < .05) and positive correlations with the serum markers β-CTX or P1NP (*P* < .05) (see Supplementary Table S3 in the Supplementary Data). After adjusting for age, HLA-C in women remained significantly negatively correlated with LS and TH BMD, while six DAPs in men, including NDUFA11, COX7A2, NDUFAB1, UQCRC1, COX5A and VDAC1, were significantly positively correlated with β-CTX (Table 2).

**Table 2:** Correlation of female and male core DAPs with BMD and BTMs (adjusted by age).

	LS BMD		FN BMD		TH BMD		β-CTX		P1NP		
	*r*	*P*	*r*	*P*	*r*	*P*	*r*	*P*	*r*	*P*	
Women											
HLA-DPB1	−0.362	.204	−0.361	.205	−0.389	.169	−0.251	.409	−0.167	.585	
HLA-DRA	−0.051	.851	0.205	.445	0.011	.968	0.082	.773	0.235	.399	
ATP6V0A1	−0.025	.937	−0.089	.772	−0.340	.255	0.183	.590	0.163	.631	
HLA-DRB1	−0.044	.891	−0.094	.771	−0.201	.532	−0.080	.815	0.029	.933	
ITGB2	0.064	.819	−0.023	.935	−0.010	.971	−0.050	.866	0.155	.596	
HLA-C	**−0.569**	**.027**	−0.111	.693	**−0.503**	**.046**	−0.194	.507	−0.174	.551	
HLA-A	−0.292	.272	0.072	.790	−0.220	.412	0.019	.946	−0.030	.914	
Men											
NDUFA11	−0.151	.606	−0.158	.589	−0.136	.643	**0.692**	**.009**	0.077	.802	
COX5B	−0.025	.925	−0.071	.786	−0.106	.685	0.430	.097	−0.132	.625	
NDUFA8	−0.255	.324	−0.143	.583	−0.120	.648	0.347	.188	−0.030	.912	
COX7A2	−0.026	.920	−0.204	.432	−0.217	.402	**0.557**	**.025**	0.058	.832	
NDUFAB1	−0.113	.676	−0.345	.190	−0.341	.196	**0.660**	**.007**	−0.075	.791	
NDUFA3	−0.125	.644	−0.254	.343	−0.262	.327	0.200	.476	0.011	.969	
UQCRC1	−0.105	.690	−0.182	.485	−0.196	.451	**0.582**	**.018**	−0.054	.842	
COX5A	−0.108	.692	−0.203	.451	−0.214	.427	**0.548**	**.034**	−0.094	.739	
NDUFV2	−0.315	.235	−0.147	.588	−0.163	.545	0.258	.354	−0.181	.518	
VDAC1	−0.174	.504	−0.174	.504	−0.152	.561	**0.517**	**.040**	0.029	.915	

Bold numbers indicate a statistically significant correlation.

ATP6V0A1, ATPase H+ transporting V0 subunit A1; BMD, Bone mineral density; BTMs, Bone turnover biomarkers; COX5A, Cytochrome c oxidase subunit 5A; COX5B, Cytochrome c oxidase subunit 5B; COX7A2, Cytochrome c oxidase subunit 7A2; DAPs, Differentially abundant proteins; FN, Femoral neck; HLA-A, Major histocompatibility complex, class I, A；HLA-C, Major histocompatibility complex, class I, C; HLA-DPB1, Major histocompatibility complex, class II, DP β1; HLA-DRA, Major histocompatibility complex, class II, DR α; HLA-DRB1, Major histocompatibility complex, class II, DR β1; ITGB2, Integrin subunit β2; LS, Lumbar spine; NDUFA3, NADH oxidoreductase subunit A3; NDUFA8, NADH oxidoreductase subunit A8; NDUFA11, NADH oxidoreductase subunit A11; NDUFAB1, NADH oxidoreductase subunit AB1; NDUFV2, NADH oxidoreductase core subunit V2; P1NP, procollagen type 1 N-peptide; TH, Total hip; UQCRC1, Ubiquinol-cytochrome c reductase core protein 1; VDAC1, Voltage-dependent anion channel 1; β-CTX, β-C-terminal telopeptide of type 1 collagen.

## Discussion

In this study, we performed a detailed proteomic analysis of valuable human skeletal specimens from patients with osteoporosis and sex-matched controls. For the first time, we identified sex-specific proteomic signatures in osteoporosis, with female patients showing enrichment of immune-inflammatory pathways and male patients exhibiting oxidative stress–related profiles. Both groups consisted of fracture patients—osteoporotic fractures in the case group and violent fractures in the control group—so any fracture-related metabolic changes were present in both cohorts and unlikely to bias between-group comparisons. These results, derived directly from bone tissue, provide unique insights into the molecular alterations in osteoporosis and reveal interactions between bone cells within their native microenvironment, which are crucial for understanding the local pathological processes of osteoporosis.

Proteomics enables the identification of numerous proteins, providing extensive insights into molecular pathways underlying bone diseases and potential therapeutic targets [2]. Hitherto, only two studies have evaluated the proteomic characteristics of bone tissue in osteoporosis patients [9, 10]. One study compared five osteoporotic patients with iron accumulation to five controls, identifying associations of GSTP1, LAMP2, COPB1 and RAB5B with bone mass and ferritin [9]. These proteins affect mainly oxidoreductase activity, GTPase activity, GTP binding and neuronal development. However, these findings, limited to iron-related osteoporosis, cannot fully explain the general osteoporosis pathogenesis. Another compared three osteopenic and three healthy subjects, reporting elevated carbonic anhydrase I and phosphoglycerate kinase 1 and reduced apolipoprotein A1 [10]. However, the fat and blood around the bone tissue were not removed, so the influence of confounding factors could not be ruled out. Our study included the largest sample size of bone samples to date and systematically analysed the DAPs in the bone tissue of female and male osteoporosis patients, which helps to elucidate the pathological processes of primary osteoporosis.

The attenuation of correlations after age adjustment, particularly in women, underscores the dominant influence of ageing on the proteomic differences observed in this study. Ageing itself is a major pathogenic factor for osteoporosis [11]. With advancing age, senescent cells accumulate within bone and marrow, releasing a senescence-associated secretory phenotype that drives chronic low-grade inflammation, promotes osteoclastogenesis and suppresses osteoblast function [12, 13]. In women, the decline of oestrogen level after menopause amplifies these immune-inflammatory pathways, particularly through the upregulation of the RANKL/TNF-α axis, thereby accelerating bone resorption [14]. After age adjustment, the associations between immune-inflammatory responses and osteoporosis in women were markedly attenuated, underscoring the dominant role of ageing as a driving factor in osteoporosis pathogenesis.

In men, ageing also exerts a profound influence on osteoporosis risk. Age-related androgen deficiency impairs the antioxidant defence system, leading to excessive ROS production and mitochondrial dysfunction [15, 16]. The resulting oxidative stress not only promotes osteoclastogenesis and suppresses osteoblast activity, but also skews mesenchymal stromal cell differentiation toward adipogenesis, increasing marrow fat and further inhibiting bone formation [17, 18]. These findings support the concept that oxidative stress is a key mediator linking ageing to skeletal fragility in men.

The proteomic profiles revealed distinct pathways between men and women, which is consistent with growing evidence of sex-specific pathophysiological mechanisms in bone fragility [8, 19]. These differences may reflect broader sex dimorphisms—women maintain a more reactive immune set point across the lifespan, whereas men exhibit higher ROS generation and vulnerability to oxidative damage. Future studies should apply sex-stratified, multi-omics profiling of human bone and marrow to dissect immune–bone crosstalk in women and mitochondrial–redox pathways in men.

This study investigated the possible pathogenesis of osteoporosis by comparing bone tissue proteomic profiles between patients with osteoporotic fractures and sex-matched controls with violent fractures. Proteomic sequencing of bone samples provided insights into the interactions among bone cells, bone marrow and the extracellular matrix under osteoporotic conditions. To our knowledge, this is the first study to examine protein expression in both female and male osteoporosis patients, highlighting sex-related differences in the pathogenesis of osteoporosis. Nevertheless, several limitations must be acknowledged. Patients with osteoporosis and healthy controls were not strictly age-matched, making it difficult to exclude potential confounding factors in older supple patients that could affect the results. Although we have addressed the age imbalance statistically by prioritising age-adjusted analyses in the main results, we have interpreted the findings with greater caution. Future studies with larger, strictly age-matched cohorts are needed to confirm these results. Furthermore, the fracture sites were obviously imbalanced between the groups, with notable skewing toward vertebral fractures in the osteoporosis group and appendicular fractures in the control group. The bone structure, function and mechanical stress differ between axial and peripheral bones, which might influence protein expression patterns [20]. While the imbalance in fracture sites might have influenced the specific proteins detected, the fundamental processes related to bone remodelling are shared between axial and peripheral bones; thus, the proteomic signatures observed in this study still offer valuable insights into the underlying molecular mechanisms of osteoporosis. Additionally, the sample size of this study was relatively small. Although intraoperative bone sampling is technically feasible in most fracture surgeries, bone specimens are not routinely collected for pathological examination unless there is suspicion of pathological fractures. Furthermore, many patients did not meet our eligibility criteria, particularly in terms of DXA-confirmed BMD status, absence of comorbidities affecting bone metabolism, and no recent use of medications known to influence bone health. In addition, participation required informed consent, and not all eligible patients agreed to enrol, further limiting recruitment. In the future, we will continue to collect more bone specimens for further in-depth study. Moreover, we excluded only patients who had received anti-osteoporosis therapy within the previous 12 months, which may have led to selection bias because of the long half-life of bisphosphonates. However, as many patients at risk of fractures are receiving medication, this is a common limitation.

In conclusion, this study identified differences in protein expression and important regulatory pathways of osteoporosis through proteomics in valuable human skeletal specimens. These findings highlight the central role of ageing and its divergent downstream mechanisms in osteoporosis pathogenesis, which may help to identify new biomarkers and key therapeutic targets for osteoporosis. We found for the first time that male and female osteoporosis patients presented with distinct proteomic results, suggesting that there are differences in the pathophysiological mechanisms of osteoporosis between females and males, which deserve further study.

## Supplementary Material

aa-25-1684-File003_afaf299

## Data Availability

The raw MS proteomics data of 40 bone tissues have been deposited in the ProteomeXchange Consortium *via* the iProX partner repository [21, 22] under Project ID PXD056587. URL: https://www.iprox.cn/page/PDV0141.html.

## References

[1] Walker MD, Shane E. Postmenopausal osteoporosis. N Engl J Med 2023;389:1979–91. 10.1056/NEJMcp2307353. 37991856

[2] Yang TL, Shen H, Liu A et al. A road map for understanding molecular and genetic determinants of osteoporosis. Nat Rev Endocrinol 2020;16:91–103. 10.1038/s41574-019-0282-7. 31792439 PMC6980376

[3] Nielson CM, Wiedrick J, Shen J et al. Identification of hip BMD loss and fracture risk markers through population-based serum proteomics. J Bone Miner Res 2017;32:1559–67. 10.1002/jbmr.3125. 28316103 PMC5489383

[4] Xu J, Cai X, Miao Z et al. Proteome-wide profiling reveals dysregulated molecular features and accelerated aging in osteoporosis: a 9.8-year prospective study. Aging Cell 2024;23:e14035. 10.1111/acel.14035. 37970652 PMC10861190

[5] Maruyama M, Rhee C, Utsunomiya T et al. Modulation of the inflammatory response and bone healing. Front Endocrinol (Lausanne) 2020;11:386. 10.3389/fendo.2020.00386. 32655495 PMC7325942

[6] International Osteoporosis Foundation: About Osteoporosis. https://www.osteoporosis.foundation/patients/about-osteoporosis. (10 October 2024, last accessed)

[7] Wang XY, Zhang RZ, Wang YK et al. An updated overview of the search for biomarkers of osteoporosis based on human proteomics. J Orthop Translat 2024;49:37–48. 10.1016/j.jot.2024.08.015. 39430131 PMC11488448

[8] Zhang YY, Xie N, Sun XD et al. Insights and implications of sexual dimorphism in osteoporosis. Bone Res 2024;12:8. 10.1038/s41413-023-00306-4. 38368422 PMC10874461

[9] Wang A, Zhang H, Li G et al. Deciphering core proteins of osteoporosis with iron accumulation by proteomics in human bone. Front Endocrinol (Lausanne) 2022;13:961903. 10.3389/fendo.2022.961903. 36313751 PMC9614156

[10] Chaput CD, Dangott LJ, Rahm MD et al. A proteomic study of protein variation between osteopenic and age-matched control bone tissue. Exp Biol Med (Maywood) 2012;237:491–8. 10.1258/ebm.2012.011374. 22619369

[11] Raisz LG, Seeman E. Causes of age-related bone loss and bone fragility: an alternative view. J Bone Miner Res 2001;16:1948–52. 10.1359/jbmr.2001.16.11.1948. 11697790

[12] Fan H, Qiao Z, Li J et al. Recent advances in senescence-associated secretory phenotype and osteoporosis. Heliyon 2024;10:e25538. 10.1016/j.heliyon.2024.e25538. 38375248 PMC10875379

[13] Li CJ, Xiao Y, Sun YC et al. Senescent immune cells release grancalcin to promote skeletal aging. Cell Metab 2021;33:1957–1973.e6. 10.1016/j.cmet.2021.08.009. 34614408

[14] Ono T, Hayashi M, Sasaki F et al. RANKL biology: bone metabolism, the immune system, and beyond. Inflamm Regen 2020;40:2. 10.1186/s41232-019-0111-3. 32047573 PMC7006158

[15] do Val Lima PR, Ronconi KS, Morra EA et al. Testosterone deficiency impairs cardiac interfibrillar mitochondrial function and myocardial contractility while inducing oxidative stress. Front Endocrinol (Lausanne) 2023;14:1206387. 10.3389/fendo.2023.1206387. 37780627 PMC10534000

[16] Shiota M, Yokomizo A, Naito S. Oxidative stress and androgen receptor signaling in the development and progression of castration-resistant prostate cancer. Free Radic Biol Med 2011;51:1320–8. 10.1016/j.freeradbiomed.2011.07.011. 21820046

[17] Tao H, Li X, Wang Q et al. Redox signaling and antioxidant defense in osteoclasts. Free Radic Biol Med 2024;212:403–14. 10.1016/j.freeradbiomed.2023.12.043. 38171408

[18] Al-Azab M, Safi M, Idiiatullina E et al. Aging of mesenchymal stem cell: machinery, markers, and strategies of fighting. Cell Mol Biol Lett 2022;27:69. 10.1186/s11658-022-00366-0. 35986247 PMC9388978

[19] Martínez de Toda I, González-Sánchez M, Díaz-Del Cerro E et al. Sex differences in markers of oxidation and inflammation. Implications for ageing. Mech Ageing Dev 2023;211:111797. 10.1016/j.mad.2023.111797. 36868323

[20] Fretwurst T, Tritschler I, Rothweiler R et al. Proteomic profiling of human bone from different anatomical sites - a pilot study. Proteomics Clin Appl 2022;16:e2100049. 10.1002/prca.202100049. 35462455

[21] Ma J, Chen T, Wu S et al. iProX: an integrated proteome resource. Nucleic Acids Res 2019;47:D1211–7. 10.1093/nar/gky869. 30252093 PMC6323926

[22] Chen T, Ma J, Liu Y et al. iProX in 2021: connecting proteomics data sharing with big data. Nucleic Acids Res 2022;50:D1522–7. 10.1093/nar/gkab1081. 34871441 PMC8728291

