# Supplementary material for: Proteomic profiling of bone tissue reveals distinct pathways in men and women with osteoporosis
Source: Age Ageing. 2025 Oct 17;54(10):afaf299. doi: 10.1093/ageing/afaf299 (PMC12531983; doi:10.1093/ageing/afaf299)

**Supplementary materials**

**Proteomic profiling of bone tissue reveals distinct pathways in men and women with osteoporosis**

**Contents**

**Supplementary Tables**

**Supplementary Table 1.** Fracture sites in female and male patients with osteoporotic or violent fractures

**Supplementary Table 2.** Relative expression of core DAPs in women and men

**Supplementary Table 3.** Correlation of female and male core DAPs with BMD and BTMs

**Supplementary Figures**

**Supplementary Figure 1.** Flow chart of patient recruitment in this study

**Supplementary Figure 2.** PCA of female and male osteoporosis and control groups

**Supplementary Figure 3.** Protein-protein interaction network analysis of key differential protein for women and men

**Supplementary Table 1. Fracture sites in female and male patients with osteoporotic or violent fractures**

| Female  (n=21) | Fracture site | Male  (n=19) | Fracture site |
| --- | --- | --- | --- |
| F_osteoporosis 01 | T8 | M_osteoporosis 01 | T8 |
| F_osteoporosis 02 | T9 | M_osteoporosis 02 | T12 |
| F_osteoporosis 03 | T11 | M_osteoporosis 03 | L1 |
| F_osteoporosis 04 | T11 | M_osteoporosis 04 | L1 |
| F_osteoporosis 05 | T12 | M_osteoporosis 05 | L2 |
| F_osteoporosis 06 | T12 | M_osteoporosis 06 | L2 |
| F_osteoporosis 07 | T12 | M_osteoporosis 07 | L3 |
| F_osteoporosis 08 | T12 | M_osteoporosis 08 | L3 |
| F_osteoporosis 09 | L1 | M_osteoporosis 09 | L4 |
| F_osteoporosis 10 | L1 | M_osteoporosis 10 | Left calcaneus |
| F_osteoporosis 11 | L2 | M_osteoporosis 11 | Left femur |
| F_osteoporosis 12 | L4 | M_osteoporosis 12 | Left tibial plateau |
| F_osteoporosis 13 | Left lateral malleolus | M_control 01 | Right clavicle |
| F_control 01 | Left femoral neck | M_control 02 | Left humerus |
| F_control 02 | Right lateral malleolus | M_control 03 | Right humerus |
| F_control 03 | Right pelvis | M_control 04 | Left lateral malleolus |
| F_control 04 | Left humerus | M_control 05 | Right tibia |
| F_control 05 | Right clavicle | M_control 06 | T12 |
| F_control 06 | Right ilium | M_control 07 | L2 |
| F_control 07 | Right fibula |  |  |
| F_control 08 | Right patella |  |  |

Abbreviations: F_osteoporosis, Female osteoporotic fracture group; F_control: Female non-osteoporotic fracture group; M_osteoporosis, Male osteoporotic fracture group; M_control, Male non-osteoporotic fracture group; T, Thoracic spine; L, Lumbar spine

**Supplementary Table 2. Relative expression of core DAPs in women and men**

| **Protein ID** | **Encoding gene** | **Protein name** | **Fold change** | **Corrected**  ***P* value** |
| --- | --- | --- | --- | --- |
| **7 core differential proteins in women** | | | | |
| P04440 | DPB1 | Major histocompatibility complex, class Ⅱ, DP β1 (HLA-DPB1) | 25.98198 | 3.14*10^-5^ |
| P01903 | DRA | Major histocompatibility complex, class Ⅱ, DR α (HLA-DRA) | 14.51862 | 1.19*10^-5^ |
| Q93050 | VPP1 | ATPase H^+^ transporting V0 subunit A1 (ATP6V0A1) | 11.10177 | 1.47*10^-5^ |
| P01911 | DRB1 | Major histocompatibility complex, class Ⅱ, DR β1 (HLA-DRB1) | 10.83317 | 0.00359704 |
| P05107 | ITB2 | Integrin subunit β2 (ITGB2) | 8.654685 | 5.63*10^-4^ |
| P10321 | HLAC | Major histocompatibility complex, class Ⅰ, C (HLA-C) | 6.856431 | 6.68*10^-5^ |
| P04439 | HLAA | Major histocompatibility complex, class Ⅰ, A (HLA-A) | 6.586219 | 1.08*10^-5^ |
| **10 core differential proteins in men** | | | | |
| Q86Y39 | NDUAB | NADH oxidoreductase subunit A11 (NDUFA11) | 5.189753 | 0.04125855 |
| P10606 | COX5B | Cytochrome c oxidase subunit 5B (COX5B) | 4.643548 | 1.17*10^-4^ |
| P51970 | NDUA8 | NADH oxidoreductase subunit A8 (NDUFA8) | 4.33606 | 5.65*10^-5^ |
| P14406 | CX7A2 | Cytochrome c oxidase subunit 7A2 (COX7A2) | 4.265426 | 8.79*10^-5^ |
| O14561 | ACPM | NADH oxidoreductase subunit AB1 (NDUFAB1) | 4.14604 | 0.00207937 |
| O95167 | NDUA3 | NADH oxidoreductase subunit A3 (NDUFA3) | 3.891459 | 0.00989594 |
| P31930 | QCR1 | Ubiquinol-cytochrome c reductase core protein 1 (UQCRC1) | 3.817026 | 7.48*10^-4^ |
| P20674 | COX5A | Cytochrome c oxidase subunit 5A (COX5A) | 3.76446 | 0.00479315 |
| P19404 | NDUV2 | NADH oxidoreductase core subunit V2 (NDUFV2) | 3.762697 | 7.74*10^-4^ |
| P21796 | VDAC1 | Voltage-dependent anion channel 1 (VDAC1) | 3.760923 | 3.83*10^-4^ |

Abbreviations: DAPs, Differentially abundant proteins

**Supplementary Table 3. Correlation of female and male core DAPs with BMD and BTMs**

|  | | LS BMD | | | | FN BMD | | | | TH BMD | | | | β-CTX | | | | P1NP | | | |
| --- | --- | --- | --- | --- | --- | --- | --- | --- | --- | --- | --- | --- | --- | --- | --- | --- | --- | --- | --- | --- | --- |
|  |  | *r* | | *P* | | *r* | | *P* | | *r* | | *P* | | *r* | | *P* | | *r* | | *P* | |
| Women | |  | |  | |  | |  | |  | |  | |  | |  | |  | |  | |
| HLA-DPB1 | | **-0.696** | | **0.004** | | **-0.747** | | **0.001** | | **-0.703** | | **0.003** | | -0.151 | | 0.606 | | 0.350 | | 0.221 | |
| HLA-DRA | | **-0.629** | | **0.007** | | **-0.597** | | **0.011** | | **-0.571** | | **0.017** | | 0.076 | | 0.779 | | **0.576** | | **0.020** | |
| ATP6V0A1 | | -0.415 | | 0.140 | | -0.491 | | 0.075 | | **-0.586** | | **0.028** | | 0.079 | | 0.806 | | 0.508 | | 0.092 | |
| HLA-DRB1 | | -0.385 | | 0.194 | | -0.477 | | 0.099 | | -0.487 | | 0.091 | | 0.033 | | 0.920 | | 0.524 | | 0.080 | |
| ITGB2 | | -0.465 | | 0.069 | | **-0.535** | | **0.033** | | -0.470 | | 0.066 | | 0.016 | | 0.956 | | **0.545** | | **0.036** | |
| HLA-C | | **-0.681** | | **0.004** | | -0.445 | | 0.084 | | **-0.646** | | **0.007** | | -0.101 | | 0.720 | | 0.264 | | 0.342 | |
| HLA-A | | **-0.674** | | **0.003** | | **-0.562** | | **0.019** | | **-0.619** | | **0.008** | | 0.037 | | 0.891 | | 0.450 | | 0.080 | |
| Men | |  | |  | |  | |  | |  | |  | |  | |  | |  | |  | |
| NDUFA11 | -0.207 | | 0.459 | | -0.265 | | 0.339 | | -0.226 | | 0.418 | | **0.709** | | **0.004** | | 0.166 | | 0.571 | |  |
| COX5B | -0.384 | | 0.115 | | **-0.592** | | **0.010** | | **-0.522** | | **0.026** | | **0.496** | | **0.043** | | 0.343 | | 0.177 | |  |
| NDUFA8 | **-0.485** | | **0.041** | | **-0.567** | | **0.014** | | **-0.483** | | **0.042** | | 0.457 | | 0.065 | | 0.342 | | 0.179 | |  |
| COX7A2 | -0.386 | | 0.114 | | **-0.648** | | **0.004** | | **-0.575** | | **0.013** | | **0.567** | | **0.018** | | 0.441 | | 0.077 | |  |
| NDUFAB1 | -0.337 | | 0.186 | | **-0.622** | | **0.008** | | **-0.588** | | **0.013** | | **0.623** | | **0.010** | | 0.204 | | 0.448 | |  |
| NDUFA3 | -0.382 | | 0.130 | | **-0.630** | | **0.007** | | **-0.587** | | **0.013** | | 0.264 | | 0.323 | | 0.324 | | 0.221 | |  |
| UQCRC1 | -0.375 | | 0.125 | | **-0.560** | | **0.016** | | **-0.507** | | **0.032** | | **0.624** | | **0.007** | | 0.300 | | 0.242 | |  |
| COX5A | -0.298 | | 0.245 | | -0.481 | | 0.050 | | -0.459 | | 0.064 | | **0.555** | | **0.026** | | 0.141 | | 0.604 | |  |
| NDUFV2 | -0.479 | | 0.052 | | **-0.503** | | **0.040** | | -0.472 | | 0.056 | | 0.310 | | 0.243 | | 0.129 | | 0.633 | |  |
| VDAC1 | -0.436 | | 0.070 | | **-0.584** | | **0.011** | | **-0.502** | | **0.034** | | **0.570** | | **0.017** | | 0.379 | | 0.133 | |  |

Bold numbers indicate a statistically significant correlation

Abbreviations: ATP6V0A1, ATPase H^+^ transporting V0 subunit A1; BMD, Bone mineral density; BTMs, Bone turnover biomarkers; COX5A, Cytochrome c oxidase subunit 5A; COX5B, Cytochrome c oxidase subunit 5B; COX7A2, Cytochrome c oxidase subunit 7A2; DAPs, Differentially abundant proteins; FN, Femoral neck; HLA-A, Major histocompatibility complex, class Ⅰ, A；HLA-C, Major histocompatibility complex, class Ⅰ, C; HLA-DPB1, Major histocompatibility complex, class Ⅱ, DP β1; HLA-DRA, Major histocompatibility complex, class Ⅱ, DR α; HLA-DRB1, Major histocompatibility complex, class Ⅱ, DR β1; ITGB2, Integrin subunit β2; LS, Lumbar spine; NDUFA3, NADH oxidoreductase subunit A3; NDUFA8, NADH oxidoreductase subunit A8; NDUFA11, NADH oxidoreductase subunit A11; NDUFAB1, NADH oxidoreductase subunit AB1; NDUFV2, NADH oxidoreductase core subunit V2; P1NP, procollagen type 1 N-peptide; TH, Total hip; UQCRC1, Ubiquinol-cytochrome c reductase core protein 1; VDAC1, Voltage-dependent anion channel 1; β-CTX, β-C-terminal telopeptide of type 1 collagen

**
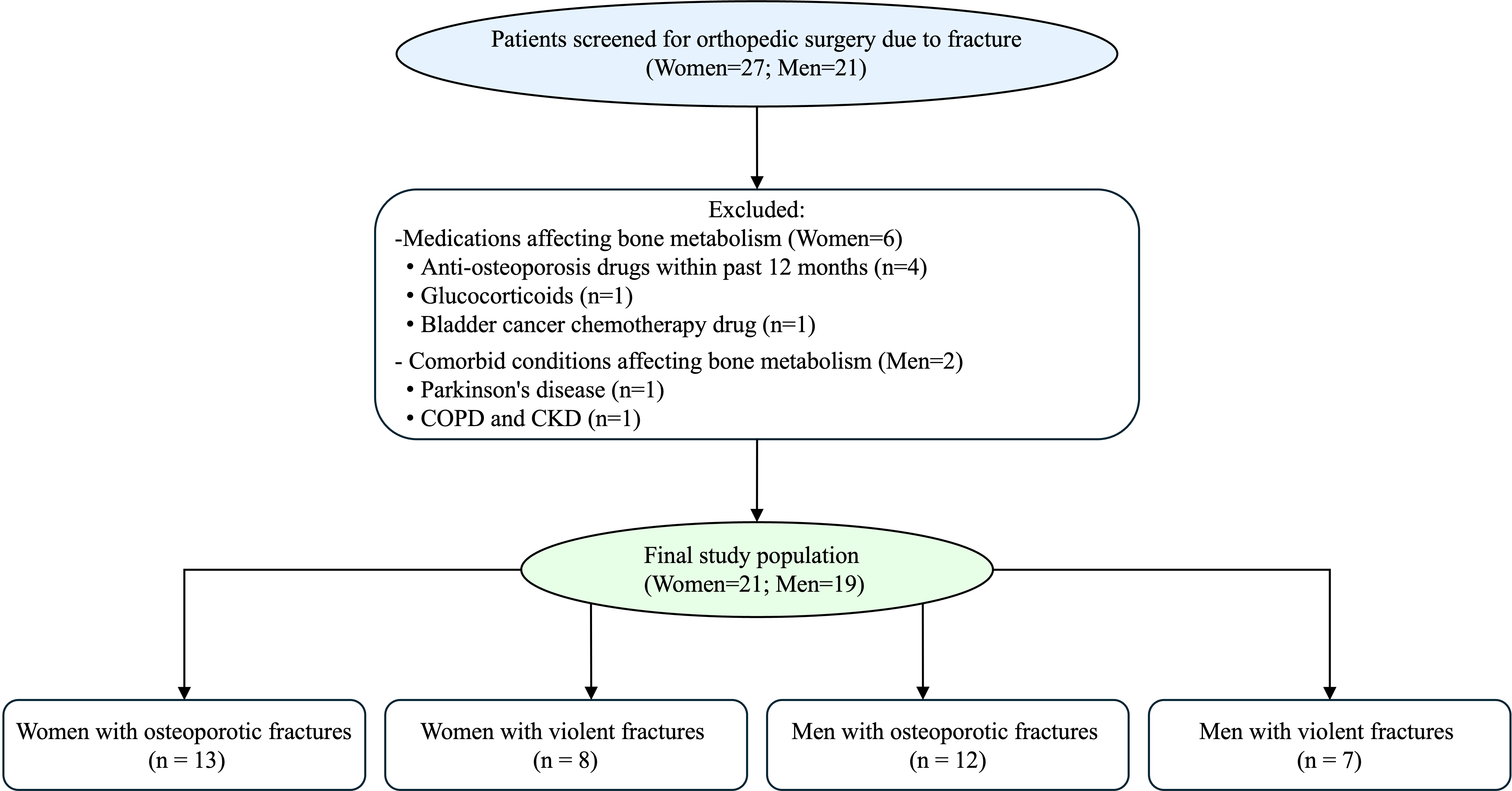
**

**Supplementary Figure 1. Flow chart of patient recruitment in this study**

COPD, Chronic obstructive pulmonary disease; CKD, Chronic kidney disease

**
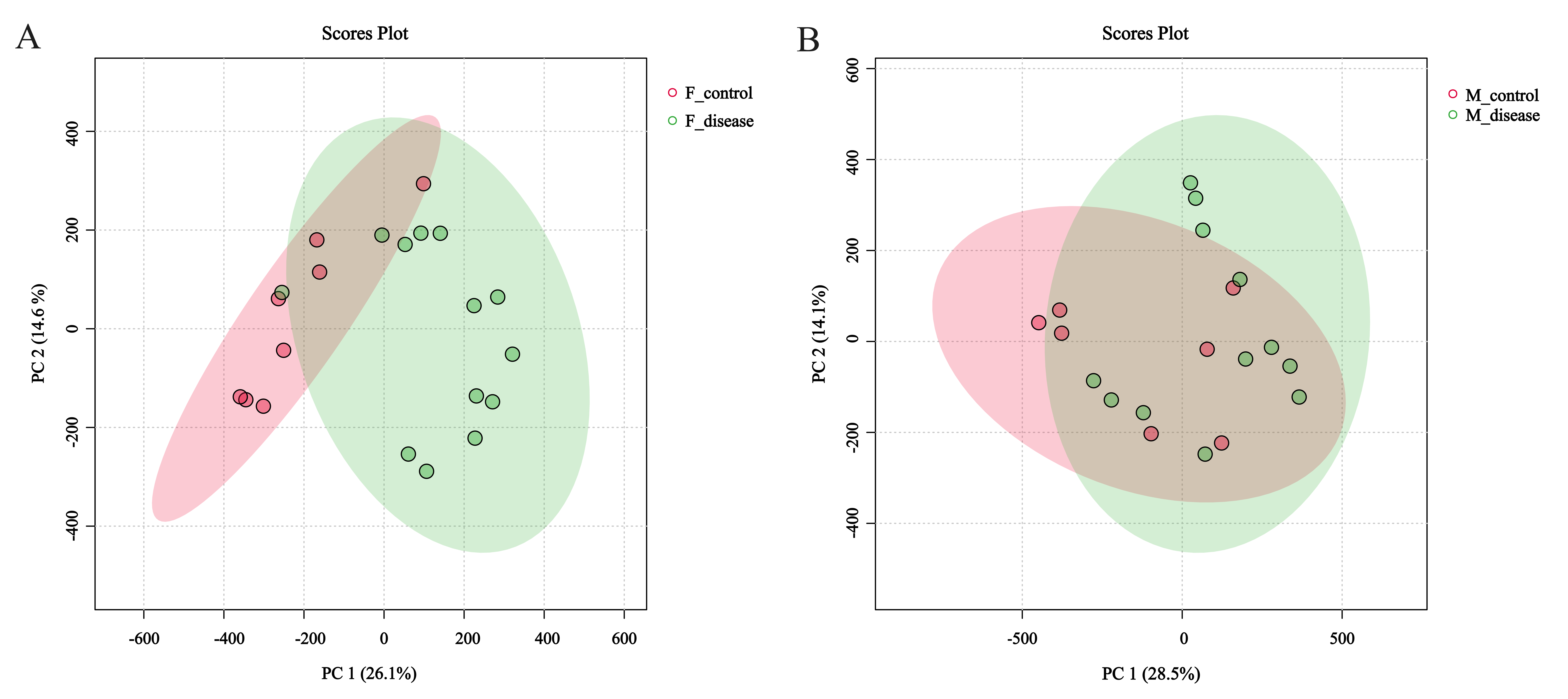
**

**Supplementary Figure 2. PCA of female and male osteoporosis and control groups**

A. PCA of female osteoporosis (green circle) and control groups (red circle)

B. PCA of male osteoporosis (green circle) and control groups (red circle)

PCA, principal component analysis


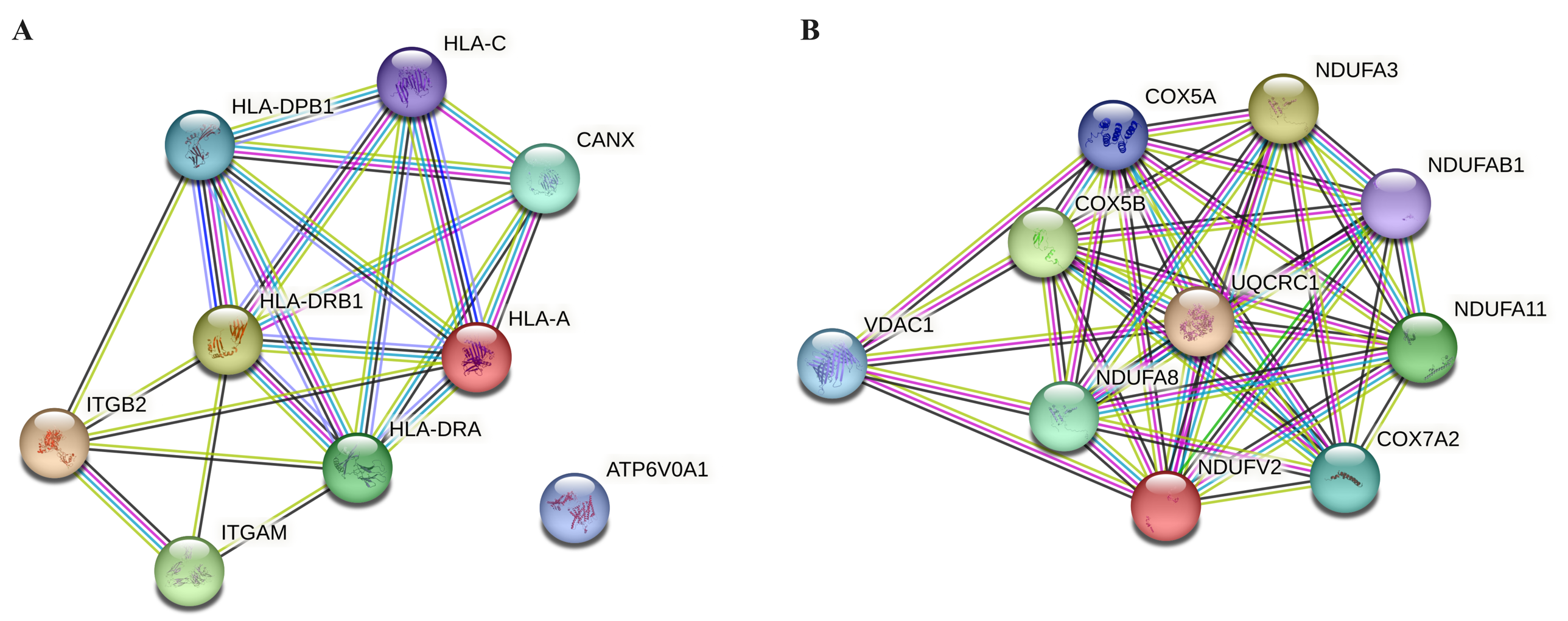


**Supplementary Figure 3. Protein-protein interaction network analysis of key differential protein for women and men**

A. Key differential protein interaction network analysis for female osteoporosis-control comparison group

B. Key differential protein interaction network diagram for male osteoporosis-control comparison group

Circles represent proteins, and lines represent the associations between proteins.

Edges:


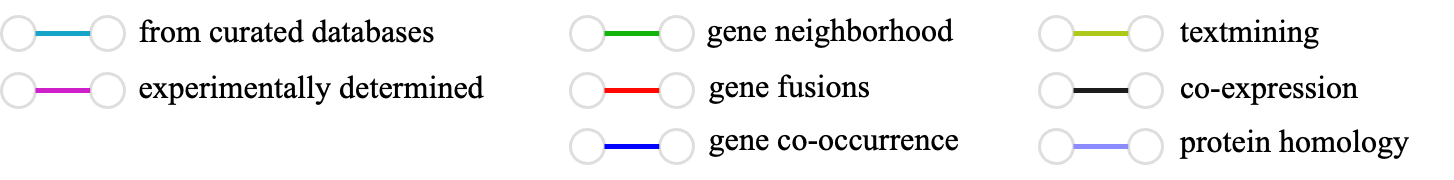

Supplement: aa-25-1684-File003_afaf299 [file aa-25-1684-file003_afaf299.docx]
